# A novel *COL2A1* mutation in a Chinese family with predominantly ocular Stickler syndrome

**DOI:** 10.3389/fgene.2025.1646923

**Published:** 2025-09-19

**Authors:** Yanyu Lin, Xin Liu, Shuxian Lin, Jiansheng Lin

**Affiliations:** ^1^ Department of Ophthalmology, Quanzhou Women’s and Children’s Hospital, Quanzhou, China; ^2^ The Affiliated Women’s and Children’s Hospital of Huaqiao University, Quanzhou, China; ^3^ Department of Laboratory Medicine, Quanzhou Women’s and Children’s Hospital, Quanzhou, China

**Keywords:** Novel mutation, COL2A1, exon 50, predominantly ocular stickler syndrome, Chinese family

## Abstract

**Purpose:**

The ocular-only variant of Stickler syndrome type I (OSTL1) is an autosomal dominant connective tissue disorder characterized by ocular abnormalities with minimal or absence of systemic involvement. This study aimed to investigate the clinical features and molecular etiology of predominantly ocular Stickler syndrome in a multigenerational pedigree.

**Methods:**

Comprehensive ophthalmic, audiological, and physical examinations were conducted on family members with predominantly ocular Stickler syndrome. Whole exome sequencing (WES) was conducted on the proband, and Sanger sequencing was used to confirm co-segregation of the identified mutation within the family.

**Results:**

Two affected individuals were identified, both presenting with myopia, megalophthalmos, retinal tears and detachment, vitreous opacification, chorioretinal scars, and early-onset cataracts. The proband’s mother had complete vision loss in her right eye. In terms of extraocular findings, the proband presented with scoliosis, and the proband’s mother had mild hearing loss in both ears. A novel likely pathogenic (LP) frameshift mutation c.3534dupT (p.Gly1179Trpfs*74) in exon 50 of the *COL2A1* was identified in both affected individuals and absent in unaffected family members. This mutation was not found in the ESP, 1000 Genomes, or EXAC databases and is predicted to cause protein truncation.

**Conclusion:**

This study reports, for the first time, the clinical manifestations associated with a novel *COL2A1* exon 50 mutation in a family with predominantly ocular Stickler syndrome. Our findings expand the known mutational spectrum of *COL2A1* and further illustrate the phenotypic variability of an ocular variant of Stickler syndrome type I with minimal systemic manifestations. These results highlight the importance of early screening in individuals at risk to enable timely diagnosis and management.

## Introduction

Stickler syndrome type I, non-syndromic ocular form (OMIM: 609508), is an autosomal dominant connective tissue disorder. This variant, also referred to as ocular-only Stickler syndrome type I (OSTL1), is characterized mainly by rhegmatogenous retinal detachment and lattice degeneration of the retina, with minimal or absence of systemic involvement (Snead et al., 2011; Richards et al., 2000a). Notably, OSTL1 shows considerable phenotypic heterogeneity among affected families (Liu et al., 2022).

The *COL2A1* gene (OMIM:120140), located on chromosome 12q13.11, is the causative gene for Stickler syndrome type I. It encodes the α1 chain of type II collagen, a structural protein composed of three α1(II) chains that is highly expressed in the vitreous body of the eye. This mutation is associated with over 15 distinct phenotypes, largely due to its role in the formation of type XI collagen, where the α1(II) peptide chain also serves as a component. Type XI collagen is expressed in different isoforms across various tissues. While its α1 chain is expressed in both ocular and cartilaginous tissues, the α2 chain is predominantly expressed in non-ocular (cartilage) tissues. Therefore, mutations affecting the α2 chain typically lead to arthropathy, with little to no impact on ocular phenotype. In contrast, mutations involving the α1 chain result in the classical form of Stickler syndrome, which affects both the ocular and skeletal structures (Snead et al., 2011).

OSTL1 is typically caused by mutations in exon 2 of *COL2A1* (Sun et al., 2020) (Tran-Viet et al., 2013). These mutations in exon 2 of *COL2A1* are associated with a fully penetrant ocular-only phenotype, characterized by membranous vitreous anomalies and megalophthalmos (Richards et al., 2000a; Richards and Snead, 2008). The phenotypic variability of exon 2 mutations has been attributed to mechanisms, such as nonsense-mediated mRNA degradation or alternative splicing (McAlinden et al., 2008). Minigene studies have confirmed that disruption of the enhancer locus in exon 2 of *COL2A1* alters the ratio between the type IIB and type IIA procollagen isoforms, which may exert adverse effects during ocular embryogenesis (McAlinden et al., 2008).

In this study, we identified a novel mutation (a non-exon 2 mutation) in the *COL2A1* gene in a family diagnosed with predominantly ocular Stickler syndrome. This study aims to explore the correlation between a novel *COL2A1* mutation and an ocular variant of Stickler syndrome type I with minimal systemic manifestations. Our findings expand the mutational spectrum of the *COL2A1* gene and provide further insights into the genotype–phenotype relationship.

## Methods

### Participants

The patients and their medical histories were identified at Quanzhou Women and Children’s Hospital. The study protocol was approved by the Ethics Committee of Quanzhou Women and Children’s Hospital. All participants provided written informed consent before inclusion in the study. All the experiments were performed in accordance with the Declaration of Helsinki and other relevant guidelines and regulations. Venous whole-blood samples were collected for molecular genetic testing.

### Clinical examination

A comprehensive clinical history was obtained for all participants, with particular attention to ophthalmic, audiological, and orthopedic conditions. The detailed ophthalmic history included the age of onset, severity, and progression of myopia, cataracts, and vitreoretinal diseases. A comprehensive ophthalmic examination was also performed, including best-corrected visual acuity assessment, slit-lamp microscopy (Suzhou 66 Vision Technology Co., Ltd., China, Model YZ5T) examination, intraocular pressure measurement using a non-contact tonometer (NIDEK, Japan, Model NT-530), fundus examination with a non-mydriatic fundus camera (Beijing Topcon Technology Development Co., Ltd., Model TRC-N), optical coherence tomography (OCT) examination (Henan Shiwei Imaging Technology Co., Ltd., Model VG200D), and ocular B-ultrasound examination using an A/B ultrasound diagnostic instrument (Tianjin Suowei Electronic Technology Co., Ltd., Model SW-2100). Individuals presenting with retinal detachment or retinal tears were classified as ‘affected.’ A general physical examination was conducted on all individuals, including an assessment of abnormalities in the face, palate, spine, joints, and hearing. The hip joint and the entire spine were examined using digital radiography. Hearing levels were measured using an audiometer.

### Whole exome sequencing (WES) analysis

Genomic DNA was extracted from the proband’s peripheral blood leukocytes. WES was performed and analyzed by BGI Genomics. Firstly, the DNA was fragmented and a library was prepared. Then, the DNA of the exons of the target genes and the adjacent splice regions were captured and enriched using the Roche KAPA HyperExome chip. Finally, the MGISEQ-2000 sequencing platform was used for mutation detection. The quality control indicators of the sequencing data are as follows: the average sequencing depth of the target region is ≥ 200×, and the proportion of the sites with an average depth in the target region > 20× is > 98.5%.

The sequenced fragments were aligned with the UCSC hg19 human reference genome using BWA, and the duplicates were removed. GATK was used for base quality value correction, SNV, INDEL and genotype detection. ExomeDepth was used for copy number variation detection at the exon level. Gene nomenclature followed the naming specifications of the Human Genome Organization Gene Nomenclature Committee (HGNC); mutation nomenclature followed the naming specifications of the Human Genome Variation Society (HGVS). Mutations were annotated and screened based on the clinical information of the tested individuals, population databases, disease databases, and bioinformatics prediction tools. The pathogenic classification of mutations was based on the sequence mutation interpretation guidelines of the American College of Medical Genetics and Genomics (ACMG) and the Association for Molecular Pathology (AMP), and referred to the detailed interpretation of these guidelines by the ClinGen Sequence Mutation Interpretation Working Group and the Association for Clinical Genomic Science (ACGS) in the United Kingdom.

### Pathogenic mutation validation and co-segregation analysis

Pathogenic mutations identified through WES were validated in all family members using Sanger sequencing. A pedigree chart was constructed to assess co-segregation of the mutation within the family.

## Results

### Clinical manifestations of the patients

**FIGURE 1:**
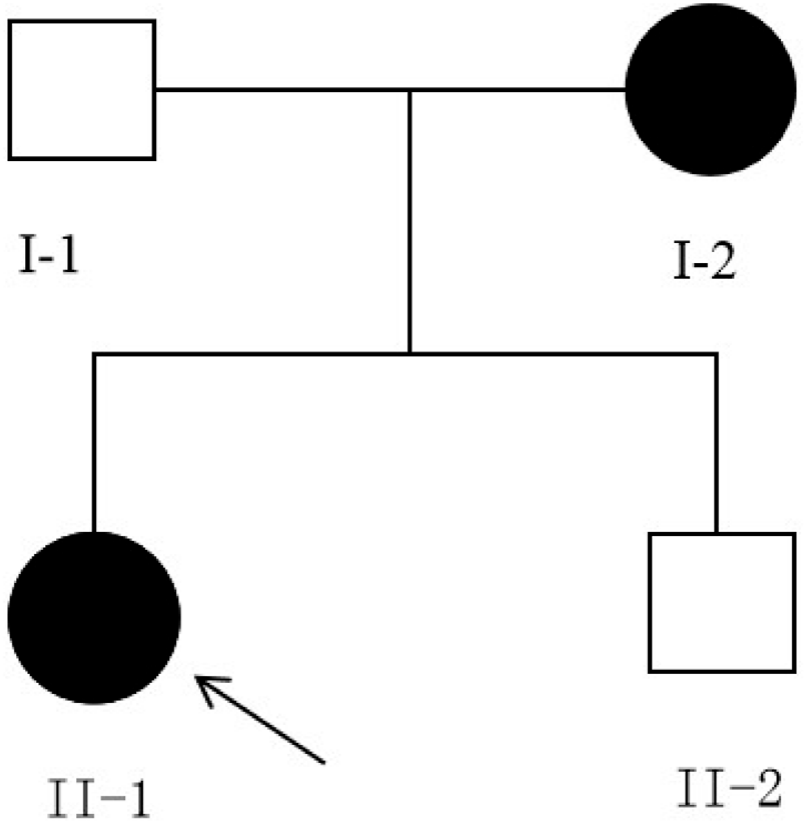
Pedigree of the family, harboring the c.3534dupT(p.Gly1179Trpfs*74) mutation of *COL2A1*. Solid symbols indicate afected individuals, and open symbols indicate unafected individuals. Arrow indicates the proband. Both affected individuals carry the heterozygous c.3534dupT (p.Gly1179Trpfs*74) mutation.

**FIGURE 2:**
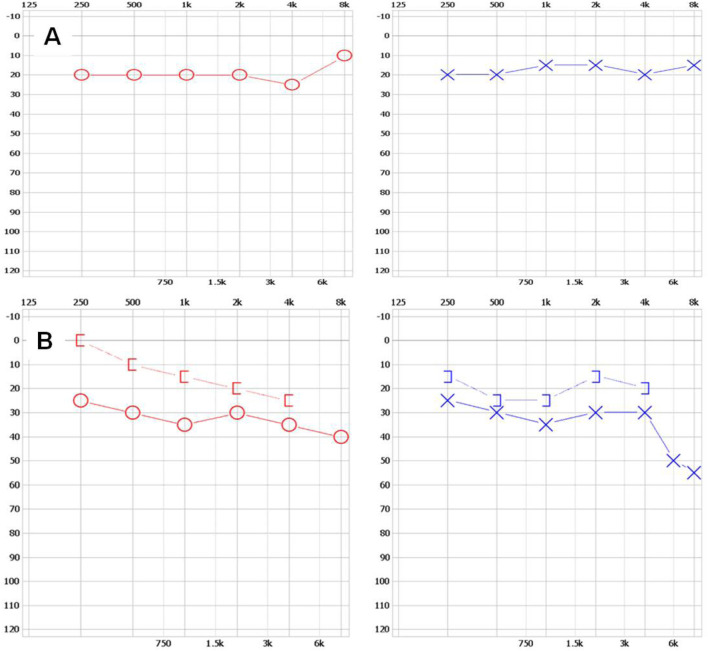
The results of hearing tests for II-1 and I-2 **(A)** The upper left and upper right respectively show the normal hearing results of the right ear and left ear of individual Ⅱ-1. **(B)** The upper left and upper right respectively show mild hearing loss in the right ear and left ear of individual Ⅰ-2. The x-axis indicates frequency (Hz); the y-axis indicates hearing level (dB).

The family with predominantly ocular Stickler syndrome reported in this study is from Fujian Province, China, and consists of two affected and two unaffected members, as shown in Figure 1. The affected members presented with myopia, megalophthalmos, retinal tears and detachment, vitreous opacification, chorioretinal scars, and early onset cataracts (Table 1). In terms of extraocular findings, the proband presented with scoliosis (Table 1), and the proband’s mother had mild hearing in both ears (Figure 2B).

**TABLE 1:** Clinical phenotype and symptoms observed in this family.

Clinical manifestations	Family members			
	Proband (II-1)	Proband’s mother (I-2)	Proband’s father (I-1)	Younger brother (II-2)
Age	18	42	45	16
Age of onset	4	CR	-	-
High myopia	yes	yes	no	no
Cataract	yes	yes	no	no
Vitreous opacification	yes	yes	no	no
retinal tear	yes	yes	no	no
Retinal detachment	yes	yes	no	no
Chorioretinal scars	yes	yes	no	no
Facial abnormalities	no	no	no	no
Scoliosis	yes	no	no	no
Flexible joint hyper-mobility	no	no	no	no
hearing loss	no	yes	no	no
Cleft palate	no	no	no	no
Mild epiphyseal dysplasia	no	no	no	no

CR stands for “can’t remember”.

Individual II-1 (the proband) developed high myopia in both eyes at age 4. By age 13, the patient had progressed to pathological myopia and underwent posterior scleral reinforcement surgery in both eyes. Ophthalmic examination showed that in terms of visual acuity (after mydriatic refraction), the visual acuity of the right eye (OD) was 0.6, and that of the left eye (OS) was 0.6. The axial length of the OD was 27.25 mm, and that of the OS was 27.48 mm, consistent with megalophthalmos. Fundus examination showed a tessellated appearance. Slit-lamp examination and ocular ultrasonography were unremarkable at that time. At age 17, the patient visited a doctor due to floaters and was diagnosed with retinal degeneration in both eyes and a retinal tear in the right eye. Retinal laser photocoagulation was performed on the right eye. Visual acuity remained at 0.6 in both eyes, and intraocular pressure measured by non-contact tonometry was 18 mmHg (OD) and 17 mmHg (OS). Fundus examination revealed two large, irregular retinal tears in the temporal and superotemporal regions of the right eye, with retinal folds along the tear margins. Membranous proliferation was observed in the temporal periphery of the left eye.

**FIGURE 3:**
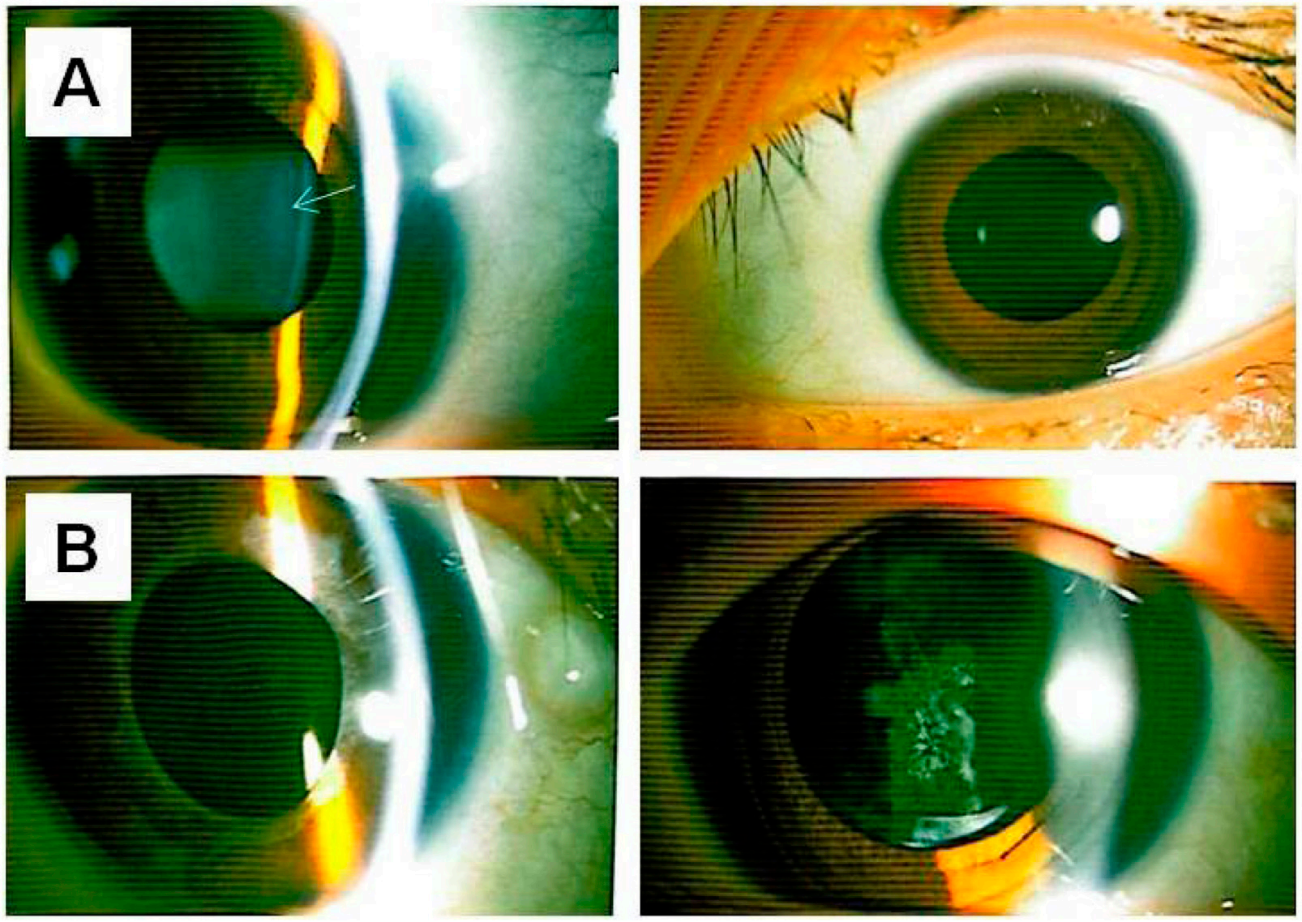
Anterior segment photographs of II-1 and I-2 **(A)** The upper left and upper right respectively show the lens opacity in the right eye and the left eye of individual II-1. **(B)** The lower left and lower right respectively show the intraocular lens in the right eye and the intraocular lens in the left eye of individual I-2 (status before laser treatment for posterior capsular opacification). The arrow indicates the lesion.

**FIGURE 4:**
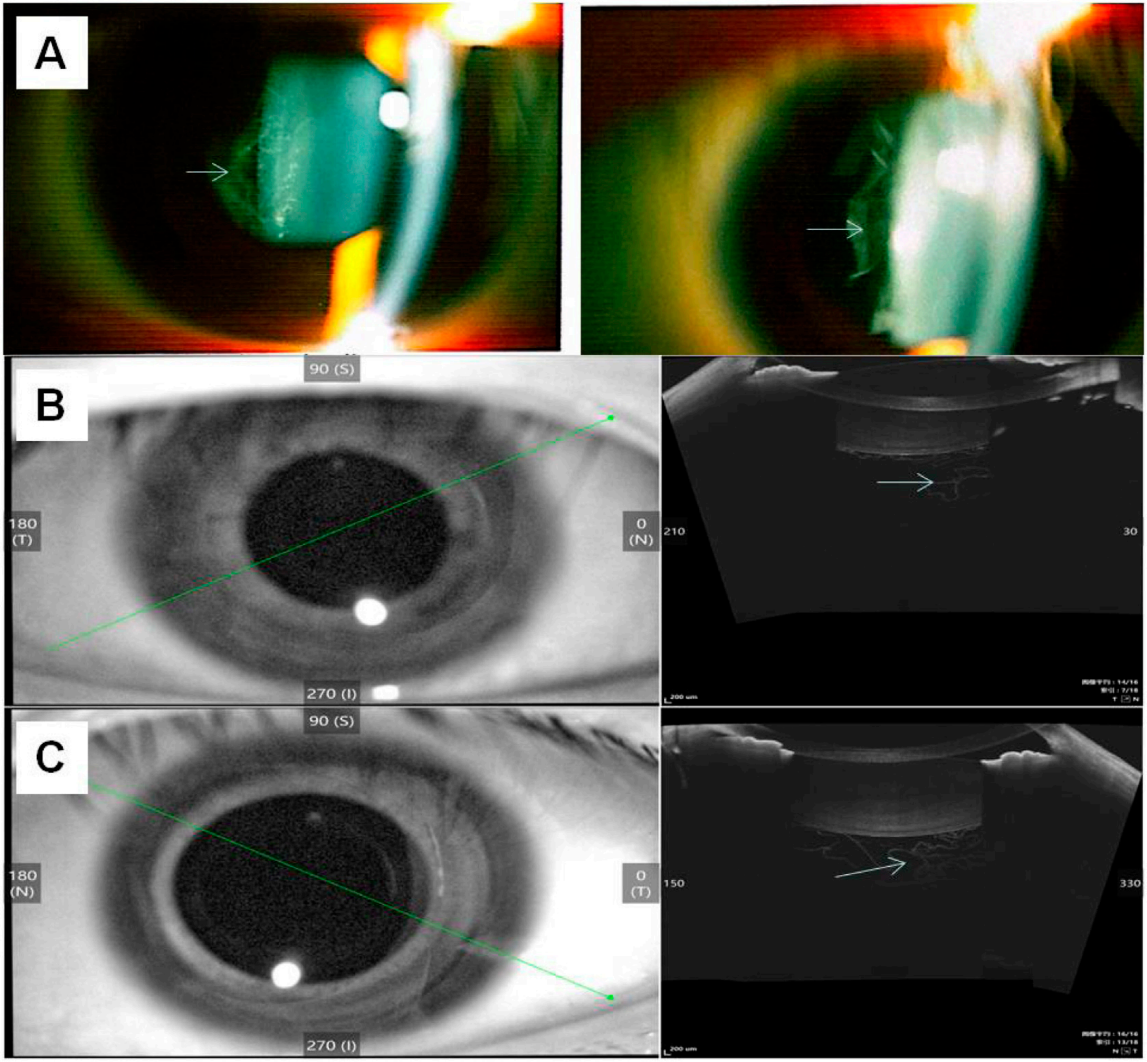
Results of image detection for membranous vitreous opacification in the individual II-1 **(A)** The left and right sides show the membranous vitreous opacities behind the lens in the right eye and left eye respectively, observed via slit-lamp examination. **(B)** OCT of the right eye shows membranous vitreous opacity; 3 **(C)** OCT of the left eye shows membranous vitreous opacity. The arrow indicates the lesion.

**FIGURE 5:**
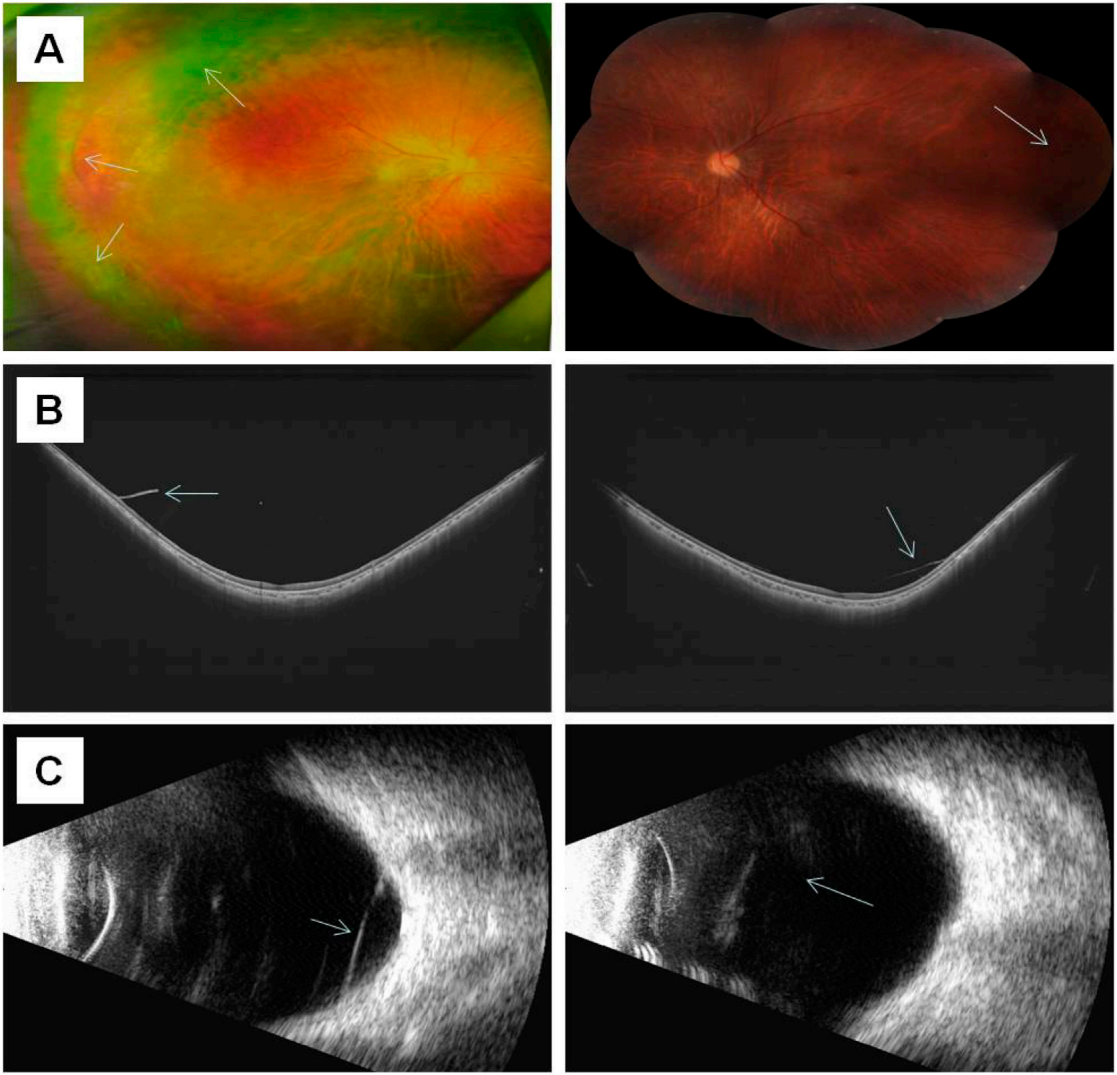
The results of ocular imaging examination of individual II-1 **(A)** The left and right sides represent the fundus images of the right eye and left eye, respectively; **(B)** The left and right sides indicate the OCT of the right eye and left eye, respectively; **(C)** The left and right sides indicate the ocular B-ultrasound examination of the right eye and left eye, respectively. The arrow indicates the lesion.

At the age of 18, the patient returned for a follow-up examination. Ophthalmic examination revealed a visual acuity of 0.6 in both eyes after mydriatic refraction. Non-contact tonometry measured intraocular pressure of 18 mmHg in the OD and 19 mmHg in the OS. Slit-lamp microscopy of the anterior segment showed decreased lens transparency in right eye, consistent with early cataract formation (Figure 3A), and the membranous vitreous opacities behind the lens in the both eyes (Figure 4A). Ocular B-ultrasound showed a retinal proliferative membrane in the right eye and vitreous opacities in the left eye (Figure 5C). OCT showed membranous vitreous opacities in both eyes (Figures 4B,C), and fibrous proliferative membranes in both eyes, while the macular structure of both eyes was normal (Figure 5B). Fundus examination of the right eye showed proliferative fibrous strands extending from the optic disc, with localized retinal blood vessels appearing stretched and straightened (Figure 5A). Multiple areas of peripheral retinal degeneration and retinal tears were also observed (Figure 5A). Laser spots were visible on the retina of the left eye (Figure 5A). There are no obvious symptoms in other systems except for scoliosis.

**FIGURE 6:**
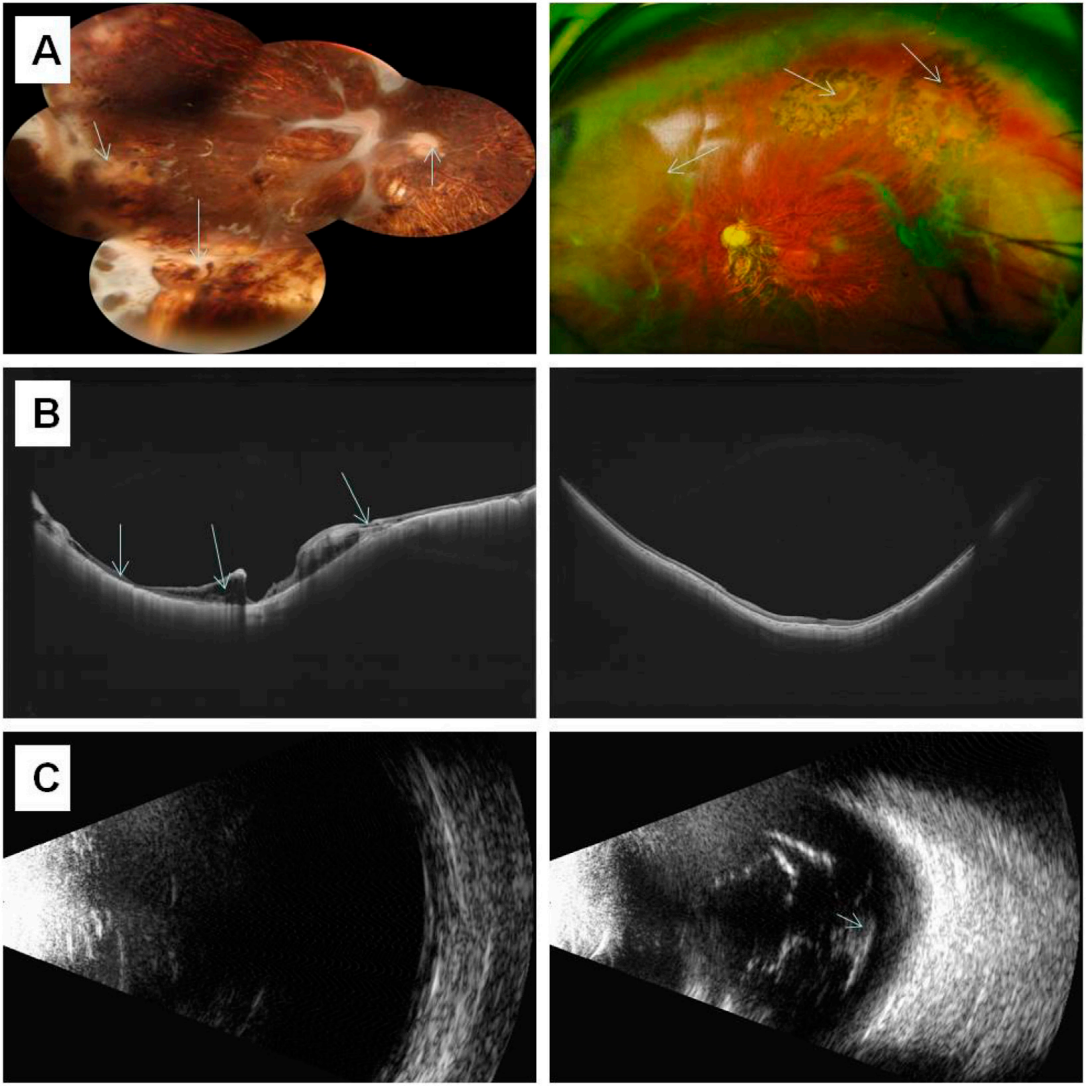
The results of ocular imaging examination of individual I-2 **(A)** The left and right sides represent the fundus images of the right eye and left eye, respectively; **(B)** The left and right sides indicate the OCT of the right eye and left eye, respectively; **(C)** The left and right sides indicate the ocular B-ultrasound examination of the right eye and left eye, respectively. The arrow indicates the lesion.

Individual I-2 (the proband’s mother) has had high myopia for several decades. She previously underwent multiple surgeries and retinal laser treatment for cataracts and retinal detachment in both eyes. At her current age of 42, the patient reported occasional floaters. Ophthalmic examination revealed no light perception in OD and a visual acuity of 0.4 in OS. Intraocular pressure was 19 mmHg in the right eye and 13 mmHg in the left eye. Axial length measurements showed 26.06 mm in the right eye and 28.81 mm in the left eye. Slit-lamp examination of the anterior segment showed intraocular lenses in both eyes, with posterior capsular opacification beneath the intraocular lens in the left eye (Figure 3B). Ocular B-ultrasound showed vitreous opacities in the left eye (Figure 6C). OCT scans demonstrated optic nerve atrophy in the right eye, with loss of normal macular structure, extensive retinal atrophy and thinning, and localized intraretinal edema (Figure 6B). The macular structure of the left eye was roughly normal, accompanied by choroidal atrophy and thinning (Figure 6B). Fundus examination showed that the right eye had pale optic nerve atrophy, extensive retinal atrophy with widespread pigment proliferation (Figure 6A). In the left eye, two retinal tears were noted in the superotemporal quadrant, surrounded by laser scars and accompanied by proliferative fibrous strands (Figure 6A). A prominent transillumination sign was observed in the superior degenerative area of the left eye, along with peripheral retinal degeneration (Figure 6A). There are no obvious symptoms in other systems except for mild hearing loss in both ears.

### Identification of a novel likely pathogenic (LP) mutation in the *COL2A1* gene for a family with predominantly ocular Stickler syndrome

**FIGURE 7:**
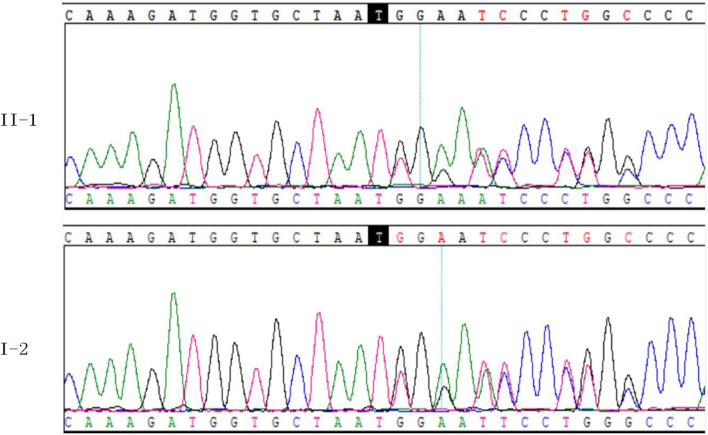
Validation of *COL2A1* NM_001844.4:c.3534dupT (p.Gly1179Trpfs*74) in II-1 and I-2.

WES was performed on the proband in this family. After mutation screening, the *COL2A1* gene was identified as the most likely candidate responsible for OSTL1. Sanger sequencing confirmed a heterozygous c.3534dupT (p.Gly1179Trpfs*74) mutation in exon 50 of the *COL2A1* gene (Figure 7). This mutation leads to a frameshift mutation that introduces a premature stop codon 74 amino acids downstream. It was not detected in control populations from the ESP, 1000 Genomes, and EXAC databases. Therefore, this mutation represents a novel LP mutation in the *COL2A1* gene that has not been previously reported.

**FIGURE 8:**
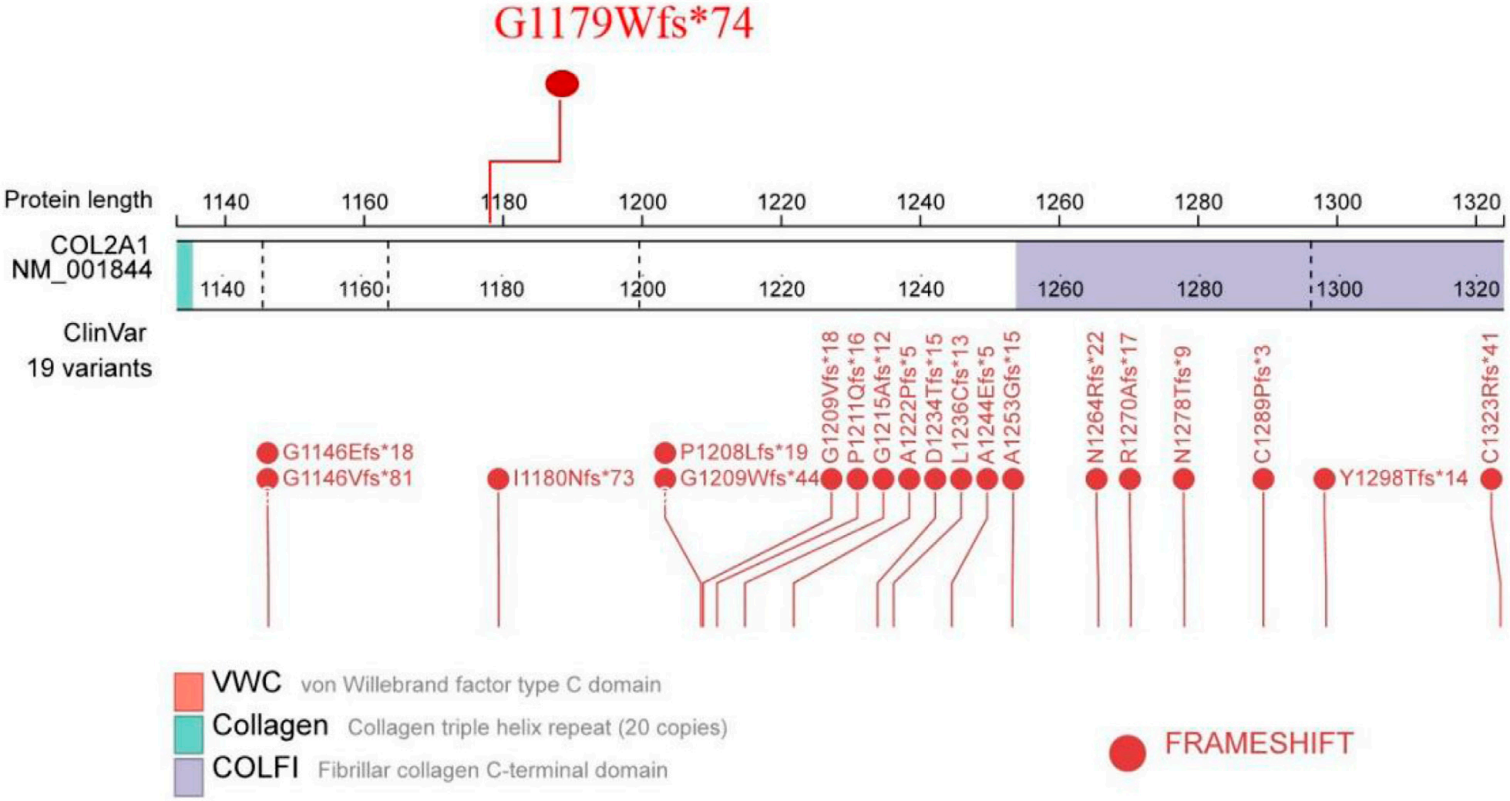
The location of the mutation site in the protein structure.

To assess co-segregation of the mutation with the disease phenotype, Sanger sequencing was performed on the remaining family members. The mutation was found to co-segregate with affected individuals, as shown in Figures 1, 7. Further analysis of the mutation’s pathogenic mechanism revealed that it disrupted the synthesis of the fibrillar collagen C-terminal domain (Figure 8), likely resulting in protein truncation. This truncation may lead to the pathogenesis of predominantly ocular Stickler syndrome.

## Discussion

Stickler syndrome is the most common cause of retinal detachment in children and the leading hereditary cause of familial retinal detachment. Unlike many other hereditary blinding eye diseases, vision loss from retinal detachment in Stickler syndrome can often be prevented with accurate diagnosis and timely prophylactic surgery (Fincham et al., 2014; Alexander et al., 2023). In this study, the proband’s mother lost vision in her right eye due to delayed diagnosis and treatment, highlighting the critical importance of prompt management. Reports indicate that there is a difference in the age of onset and penetrance among patients with OSTL1 and *COL2A1* mutations (Tompson et al., 2017). In families with OSTL1, the penetrance of vitreoretinal degeneration in affected patients exceeds 90% by age 20 (Parma et al., 2002; Donoso et al., 2003). Currently, the overall severity of ocular disease in the proband is milder than that of her mother. This phenotypic and age-of-onset variability in patients with predominantly ocular Stickler syndrome may pose challenges for accurate clinical diagnosis. OSTL1 is not absolutely devoid of extraocular symptoms; it is just that the extraocular symptoms are relatively mild. The OSTL1 family has also been reported to have some extraocular symptoms, such as sensorineural hearing loss, midline clefts, and joint abnormalities (Richards et al., 2000a; Richards et al., 2006). In addition to ocular symptoms, the proband in this study was found to have scoliosis, and the proband’s mother had mild hearing loss. Therefore, a comprehensive understanding of the ocular and systemic manifestations associated with Stickler syndrome is crucial for accurate diagnosis and effective management.

The prominent features of OSTL1 are congenital membranous vitreous anomalies, retinal detachment, and congenital megalophthalmos (Fincham et al., 2014). Both affected individuals in this study exhibited these characteristic ocular findings. Previous reports indicate that the age of onset for retinal detachment in OSTL ranges from 6 to 27 years (Go et al., 2003), consistent with the proband’s presentation at age 17. In a large OSTL1 pedigree, pre-senile cataracts were observed in up to 78% of affected individuals (Parma et al., 2002). The characteristic quadrantic lamellar lens opacity can be helpful in alerting to the possible diagnosis, particularly in sub-groups with an ocular-only phenotype (Snead et al., 2024). Notably, both affected individuals in this study had early-onset cataracts. Paying close attention to the natural course of early-onset cataracts is also crucial for identifying patients with more subtle symptoms.

Missense and nonsense mutations in the alternatively spliced exon 2 of *COL2A1* are known to cause OSTL1 (McAlinden et al., 2008). Additionally, OSTL1 has been reported to result from missplicing caused by mutations in introns 47 and 51 (Richards et al., 2006; Yoon et al., 2016). Interestingly, the Arg453Ter mutation in exon 30 of *COL2A1*, which was previously reported in patients with classic Stickler syndrome, has also been associated with OSTL1 (Go et al., 2003). In this study, we identified a novel LP mutation in exon 50 of *COL2A1,* which is rare compared to the more commonly affected exon 2. These research findings suggest that attention should be paid to mutations in non-exon 2 regions. Although individual report had linked a deep intronic COL2A1 mutation to a family with early-onset high myopia/ocular-only Stickler syndrome (Sun et al., 2020), WES remains the primary method for the early diagnosis of predominantly ocular Stickler syndrome. In patients with Stickler syndrome, the majority of mutations in the *COL2A1* gene that lead to a premature termination codon trigger the nonsense-mediated mRNA decay (NMD) mechanism, ultimately causing haploinsufficiency of the *COL2A1* gene and thereby affecting collagen synthesis (Richards et al., 2000b; Freddi et al., 2000). However, the phenotypic diversity in a family with OSTL1 suggests that factors like NMD efficiency and genetic modifier factors can regulate the severity of the disease (Go et al., 2003). The mutations being close to the C terminus are likely less severe compared to mutations near the N terminus or at the beginning of the triple helix as the majority of the protein is intact (Zhang et al., 2020; Soh et al., 2022). The c.3641delC frameshift mutation in exon 51 results in high myopia and associated vitreous and retinal lesions (predominantly ocular) (Jacobson et al., 2023), because this mutation causes a frameshift that affects the last portion of the triple helix domain and the C terminus (Zhang et al., 2020). The frameshift mutation in exon 50 in this study, which causes a disease phenotype with predominantly ocular manifestations is similar to the c.3641delC frameshift mutation in exon 51 that causes a disease phenotype predominantly involving the eyes.

This study was based on a specific family, and the samples may have a biased genetic background. Additionally, there is a lack of long-term follow-up data on ocular lesions, such as the progression rate of early-onset cataracts, the recurrence risk of retinal detachment, and the long-term prognosis after surgery. This makes it difficult to fully clarify the natural course of the disease and the long-term effects of intervention measures.

## Conclusion

In summary, affected members of the predominantly ocular Stickler syndrome family exhibited clinical variability. In this study, we identified a novel non-exon 2 LP mutation in the *COL2A1* gene in affected members. Our findings expand the mutational spectrum of *COL2A1* and enhance the understanding of the phenotypic variability associated with an ocular variant of Stickler syndrome type I with minimal systemic manifestations.

## Data Availability

The raw sequence data reported in this article have been deposited in the Genome Sequence Archive (Genomics, Proteomics & Bioinformatics 2021) in National Genomics Data Center (Nucleic Acids Res 2022), China National Center for Bioinformation/Beijing Institute of Genomics, Chinese Academy of Sciences (GSA-Human: HRA013257) that are publicly accessible at https://ngdc.cncb.ac.cn/gsa-human.

## Abbreviations

OSTL1, ocular-only Stickler syndrome type I; WES, whole exome sequencing; LP, likely pathogenic; OD, right eye; OS, left eye; OCT, optical coherence tomography; NMD, nonsense-mediated mRNA decay.

## References

[B1] Alexander P. Fincham G. S. Brown S. Collins D. McNinch A. M. Poulson A. V. (2023). Cambridge prophylactic protocol, retinal detachment, and Stickler syndrome. N. Engl. J. Med*.* 388, 1337–1339. 10.1056/NEJMc2211320 37018499

[B2] Donoso L. A. Edwards A. O. Frost A. T. Ritter R. Ahmad N. Vrabec T. (2003). Clinical variability of Stickler syndrome: role of exon 2 of the collagen COL2A1 gene. Surv. Ophthalmol*.* 48, 191–203. 10.1016/s0039-6257(02)00460-5 12686304

[B3] Fincham G. S. Pasea L. Carroll C. McNinch A. M. Poulson A. V. Richards A. J. (2014). Prevention of retinal detachment in Stickler syndrome: the Cambridge prophylactic cryotherapy protocol. Ophthalmology 121, 1588–1597. 10.1016/j.ophtha.2014.02.022 24793526

[B4] Freddi S. Savarirayan R. Bateman J. F. (2000). Molecular diagnosis of Stickler syndrome: a COL2A1 stop codon mutation screening strategy that is not compromised by mutant mRNA instability. Am. J. Med. Genet. 90, 398–406. 10.1002/(sici)1096-8628(20000228)90:5<398::aid-ajmg10>3.3.co;2-z 10706362

[B5] Go S. L. Maugeri A. Mulder J. J. van Driel M. A. Cremers F. P. M. Hoyng C. B. (2003). Autosomal dominant rhegmatogenous retinal detachment associated with an Arg453Ter mutation in the COL2A1 gene. Invest Ophthalmol. Vis. Sci*.* 44, 4035–4043. 10.1167/iovs.02-0736 12939326

[B6] Jacobson A. Besirli C. G. Bohnsack B. L. (2023). Characteristics of a three-generation family with Stickler syndrome type I carrying two different COL2A1 mutations. Genes (Basel) 14, 847. 10.3390/genes14040847 37107605 PMC10138194

[B7] Liu X. Dong H. Gong Y. Wang L. Zhang R. Zheng T. (2022). A novel missense mutation of COL2A1 gene in a large family with Stickler syndrome type I. J. Cell Mol. Med. 26, 1530–1539. 10.1111/jcmm.17187 35064646 PMC8899160

[B8] McAlinden A. Majava M. Bishop P. N. Perveen R. Black G. C. M. Pierpont M. E. (2008). Missense and nonsense mutations in the alternatively-spliced exon 2 of COL2A1 cause the ocular variant of Stickler syndrome. Hum. Mutat. 29, 83–90. 10.1002/humu.20603 17721977

[B9] Parma E. S. Körkkö J. Hagler W. S. Ala-Kokko L. (2002). Radial perivascular retinal degeneration: a key to the clinical diagnosis of an ocular variant of Stickler syndrome with minimal or no systemic manifestations. Am. J. Ophthalmol*.* 134, 728–734. 10.1016/s0002-9394(02)01646-x 12429250

[B10] Richards A. J. Snead M. P. (2008). The influence of pre-mRNA splicing on phenotypic modification in Stickler's syndrome and other type II collagenopathies. . 22, 1243–1250. 10.1038/eye.2008.34 18309338

[B11] Richards A. J. Martin S. Yates J. R. Scott J. D. Baguley D. M. Pope F. M. (2000a). COL2A1 exon 2 mutations: relevance to the stickler and wagner syndromes. Br. J. Ophthalmol. 84, 364–371. 10.1136/bjo.84.4.364 10729292 PMC1723423

[B12] Richards A. J. Baguley D. M. Yates J. R. Lane C. Nicol M. Harper P. S. (2000b). Variation in the vitreous phenotype of Stickler syndrome can be caused by different amino acid substitutions in the X position of the type II collagen Gly-X-Y triple helix. Am. J. Hum. Genet. 67, 1083–1094. 10.1016/S0002-9297(07)62938-3 11007540 PMC1288550

[B13] Richards A. J. Laidlaw M. Whittaker J. Treacy B. Rai H. Bearcroft P. (2006). High efficiency of mutation detection in type 1 Stickler syndrome using a two-stage approach: vitreoretinal assessment coupled with exon sequencing for screening COL2A1. Hum. Mutat. 27, 696–704. 10.1002/humu.20347 16752401

[B14] Snead M. P. McNinch A. M. Poulson A. V. Bearcroft P. Silverman B. Gomersall P. (2011). Stickler syndrome, ocular-only variants and a key diagnostic role for the ophthalmologist. Eye (Lond). 25, 1389–1400. 10.1038/eye.2011.201 21921955 PMC3213659

[B15] Snead M. P. Lovicu F. J. Nixon T. R. Richards A. J. Martin H. (2024). Pathobiology of the crystalline lens in Stickler syndrome. Prog. Retin Eye Res*.* 103, 101304. 10.1016/j.preteyeres.2024.101304 39349161

[B16] Soh Z. Richards A. J. McNinch A. Alexander P. Martin H. Snead M. P. (2022). Dominant Stickler syndrome. Genes (Basel) 13, 1089. 10.3390/genes13061089 35741851 PMC9222743

[B17] Sun W. Xiao X. Li S. Jia X. Zhang Q. (2020). A novel deep intronic COL2A1 mutation in a family with early-onset high myopia/ocular-only Stickler syndrome. Ophthalmic Physiol. Opt*.* 40, 281–288. 10.1111/opo.12682 32196734

[B18] Tompson S. W. Johnson C. Abbott D. Bakall B. Soler V. Yanovitch T. L. (2017). Reduced penetrance in a large Caucasian pedigree with Stickler syndrome. Ophthalmic Genet. 38 (1), 43–50. 10.1080/13816810.2016.1275018 28095098 PMC6680000

[B19] Tran-Viet K. N. Soler V. Quiette V. Powell C. Yanovitch T. Metlapally R. (2013). Mutation in collagen II alpha 1 isoforms delineates stickler and wagner syndrome phenotypes. Mol. Vis. 19, 759–766. 23592912 PMC3626300

[B20] Yoon J. M. Jang M. A. Ki C. S. Kim S. J. (2016). Two likely pathogenic variants of COL2A1 in unrelated Korean patients with ocular-only variants of Stickler syndrome: the first molecular diagnosis in Korea. Ann. Lab. Med. 36, 166–169. 10.3343/alm.2016.36.2.166 26709265 PMC4713851

[B21] Zhang B. Zhang Y. Wu N. Li J. Liu H. Wang J. (2020). Integrated analysis of COL2A1 variant data and classification of type II collagenopathies. Clin. Genet. 97, 383–395. 10.1111/cge.13680 31758797

